# Preference for daily (1HP) vs. weekly (3HP) isoniazid-rifapentine among people living with HIV in Uganda

**DOI:** 10.5588/ijtldopen.23.0283

**Published:** 2024-02-01

**Authors:** A. Musinguzi, H. E. Aschmann, J. L. Kadota, J. Nakimuli, F. Welishe, J. Kakeeto, C. Namale, L. Akello, A. Nakitende, C. Berger, A. Katamba, J. Tumuhamye, N. Kiwanuka, D. W. Dowdy, A. Cattamanchi, F. C. Semitala

**Affiliations:** ^1^ Infectious Diseases Research Collaboration, Kampala,; ^2^ School of Public Health, Makerere University College of Health Sciences, Kampala, Uganda;; ^3^ Division of Pulmonary and Critical Care Medicine, San Francisco General Hospital, University of California San Francisco, San Francisco, CA, USA; ^4^ Department of Epidemiology and Biostatistics, and; ^5^ Center for Tuberculosis, San Francisco General Hospital, University of California San Francisco, San Francisco, CA, USA;; ^6^ Uganda Tuberculosis Implementation Research Consortium, Walimu, Kampala, Uganda;; ^7^ Clinical Epidemiology & Biostatistics Unit, Department of Medicine, Makerere University College of Health Sciences, Kampala, Uganda;; ^8^ Department of Epidemiology, Johns Hopkins Bloomberg School of Public Health, Baltimore, MD, USA;; ^9^ Division of Pulmonary Diseases and Critical Care Medicine, University of California Irvine, Irvine, CA, USA;; ^10^ Department of Medicine, Makerere University College of Health Sciences, Kampala, Uganda; ^11^ Makerere University Joint AIDS Program, Kampala, Uganda

**Keywords:** tuberculosis preventive treatment, acceptability, values, human immunodeficiency virus

## Abstract

**BACKGROUND:**

Both 1 month of daily (1HP) and 3 months of weekly (3HP) isoniazid-rifapentine are recommended as short-course regimens for TB prevention among people living with HIV (PLHIV). We aimed to assess acceptability and preferences for 1HP vs. 3HP among PLHIV.

**METHODS:**

In a cross-sectional survey among PLHIV at an HIV clinic in Kampala, Uganda, participants were randomly assigned to a hypothetical scenario of receiving 1HP or 3HP. Participants rated their level of perceived intention and confidence to complete treatment using a 0–10 Likert scale, and chose between 1HP and 3HP.

**RESULTS:**

Among 429 respondents (median age: 43 years, 71% female, median time on ART: 10 years), intention and confidence were rated high for both regimens. Intention to complete treatment was rated at least 7/10 by 92% (189/206 randomized to 1HP) and 93% (207/223 randomized to 3HP). Respectively 86% (178/206) and 93% (208/223) expressed high confidence to complete treatment. Overall, 81% (348/429) preferred 3HP over 1HP.

**CONCLUSIONS:**

Both 1HP and 3HP were highly acceptable regimens, with 3HP preferred by most PLHIV. Weekly, rather than daily, dosing appears preferable to shorter duration of treatment, which should inform scale-up and further development of short-course regimens for TB prevention.

## RÉSUMÉ

**CONTEXTE:**

L'association isoniazide-rifapentine est recommandée comme traitement de courte durée pour la prévention de la TB chez les personnes vivant avec le VIH (PVVIH), à raison d'un mois de traitement quotidien (1HP) et de 3 mois de traitement hebdomadaire (3HP). Nous avons cherché à évaluer l'acceptabilité et les préférences des PVVIH pour le traitement 1HP par rapport au traitement 3HP.

**MÉTHODES:**

Dans le cadre d'une enquête transversale menée auprès de PVVIH dans une clinique VIH de Kampala, en Ouganda, les participants ont été assignés de manière aléatoire à un scénario hypothétique de réception de 1HP ou de 3HP. Les participants ont évalué leur niveau d'intention perçue et de confiance pour terminer le traitement en utilisant une échelle de Likert de 0 à 10 et ont choisi entre 1HP et 3HP.

**RÉSULTATS:**

Parmi les 429 répondants (âge médian : 43 ans, 71% de femmes, durée médiane de la thérapie antirétrovirale : 10 ans), l'intention et la confiance ont été jugées élevées pour les deux schémas. L'intention de terminer le traitement a été évaluée à au moins 7/10 par 92% (189/206 randomisés pour 1HP) et 93% (207/223 randomisés pour 3HP). Respectivement 86% (178/206) et 93% (208/223) ont exprimé une grande confiance dans le fait de terminer le traitement. Dans l'ensemble, 81% (348/429) ont préféré la 3HP à la 1HP.

**CONCLUSION:**

Les schémas 1HP et 3HP étaient tous deux très acceptables, le schéma 3HP étant préféré par la plupart des PVVIH. L'administration hebdomadaire, plutôt que quotidienne, semble préférable à une durée de traitement plus courte, ce qui devrait inspirer l'extension et le développement de schémas thérapeutiques de courte durée pour la prévention de la TB.

Improving uptake for TB preventive treatment (TPT) is crucial to address the TB pandemic; however, there is little evidence on treatment preferences among people living with HIV (PLHIV) in high TB incidence settings.^1,2^ Although 6 months of isoniazid preventive treatment (IPT) has been accessible for decades, shorter-course TPT is not widely available in routine care.^3,4^ Recently, 3 months of weekly isoniazid-rifapentine (3HP) has been recommended in many high TB burden countries, including Uganda, while 1 month of daily isoniazid-rifapentine (1HP), another WHO-recommended short-course TPT regimen, is not yet available.^5^ These newer, shorter TPT regimens have similar efficacy, higher treatment completion rates, and a lower incidence of serious adverse events than the traditional 6–9 months of IPT.^6–8^ In addition, the cost of rifapentine has dropped since 2019, and fixed-dose combinations have become available, making 1HP and 3HP viable alternatives to IPT.^3,9,10^

Despite the emphasis on patient-centered care, which takes into account the preferences and values of PLHIV in the WHO End TB strategy,^11^ it remains uncertain whether patients would prefer 3HP or 1HP. Patient preferences could inform TPT regimen recommendations and scale-up, but there may be trade-offs with cost and the feasibility of offering several regimens.^12^ Treatment preference impacts patient adherence, satisfaction, and outcomes.^13^ Evidence on TPT preferences among PLHIV is especially sparse.^2^ Studies in low TB incidence settings found that TPT regimens with higher effectiveness, fewer side effects and shorter duration were preferred.^14,15^ Prior work to inform TPT policy has measured perceived confidence to complete treatment (self-efficacy),^16^ which refers to people’s beliefs about their individual capabilities to execute behaviors necessary to achieve important goals.^17^ In addition, perceived intention (commitment to act in a certain way) is influenced by self-efficacy, as well as patients’ attitudes and subjective norms.^18^ Thus, individual perceptions of confidence and intention reflect treatment acceptability and are strong predictors of behavior.^16–18^ Therefore, in this cross-sectional survey, we aimed to estimate the acceptability of 1HP and 3HP among adult PLHIV in a high TB incidence setting using perceived confidence and intention, as well as preferences between the two regimens, to inform public health decisions.

## METHODS

### Study design and participants

We conducted a cross-sectional, randomized, non-interventional survey among adult PLHIV (age ≥18 years) attending the Mulago Immune Suppression Syndrome (ISS) clinic under the Makerere University Joint AIDS Program (MJAP) between July and November 2022. Mulago ISS clinic is the largest specialized outpatient HIV/AIDS clinic in Uganda, with over 16,000 clients active in care. In 2022, 5,019 PLHIV initiated TPT at the clinic; 79% received IPT and 21% 3HP. Mulago ISS clinic started using 3HP in July 2020 through the 3HP Options Trial (clinicaltrials.gov NCT03934931), a type 3 effectiveness-implementation trial of optimized 3HP delivery strategies.^19^ In June 2022, 3HP was rolled out at the clinic as part of routine national HIV programming. Although recommended by national HIV guidelines,^5^ 1HP was not in use at the clinic when this study was conducted.

We invited consecutive adult PLHIV attending their routine HIV/AIDS clinic appointments who had not started TPT within the past year, were not currently being treated for active TB, and were not incarcerated to participate in the survey. All interested and eligible PLHIV were included. All participants provided written informed consent in English or Luganda. Given that many clients had previously taken TPT, excluding them may have led to selection bias. Moreover, retreatment with TPT could be considered in the future. Thus, we included PLHIV with prior TPT (3HP or IPT) experience who had not been treated within the last year.

### Data collection

Participants were randomly assigned (using simple randomization) to hypothetical scenarios of receiving either 1HP or 3HP via the in-built randomization feature of Sawtooth’s Lighthouse studio offline survey app, which also facilitated survey data collection.^20^ Allocation was concealed for the first part of the survey until the interviewer had to present either 1HP or 3HP information to elicit confidence and intention for the respective regimen. Participant education flipbooks, available in English and Luganda, were used to inform participants about TB, latent TB infection (LTBI), as well as 1HP or 3HP regimen-specific information on efficacy, number of tablets per dose, dosing frequency, duration of treatment, potential drug–drug interactions, including with antiretroviral therapy (ART), and potential side effects (Supplementary Data 1). Participant education materials and the questionnaire design were refined based on structured feedback from the first 29 participants and interviewers during a pilot phase. This feedback led to minor word changes and a change in the order of questions. All data were included in the final analysis.

Following this education, participants were asked to rate their level of perceived confidence and intention to complete the assigned hypothetical regimen, using a visual 11-point Likert scale ranging from ‘0’ (strongly disagree) to ‘10’ (strongly agree). Participants assigned to 1HP were asked to respond to the following standard statements: “I feel confident that I would be capable of completing all 28 daily doses of this treatment” and “I would intend to complete all 28 daily doses of this treatment”. Participants assigned to 3HP responded to equivalent statements with 12 weekly doses. After rating confidence and intention for one regimen, participants were given a brief comparison of key features to the alternative regimen (1HP or 3HP) using illustrations in the participant education flipbook. They were then asked to state their preference between 1HP and 3HP.

Participants’ sociodemographic and clinical baseline characteristics were self-reported (age, sex, education level, employment status, current use of hormonal contraception) or extracted from electronic medical records (ART status, regimen, and duration, viral load status, prior TB and prior TPT). Participants’ self-reported age was confirmed using electronic medical records. Participants also self-reported household characteristics with which we derived the global multidimensional poverty index (MPI) – a measure of poverty that examines deprivations across 10 indicators in the dimensions of health, education, and standards of living; people deprived in one-third or more of all indicators are considered multidimensionally poor.^21^ The questionnaire was administered by six trained Ugandan research assistants (JN, FW, JK, CN, LA, and AN), who were fluent in both English and Luganda. They reviewed flipbook content with participants and encouraged and answered any questions prior to administering the survey.

### Outcomes

The main outcomes were 1) confidence and 2) intention to complete 1HP or 3HP (11-point Likert scale), and 3) preference for 1HP compared to 3HP (binary). We explored associations with sociodemographic and clinical characteristics. Participants’ confidence and intention were also considered predictors of preference for 1HP vs. 3HP.

### Statistical analysis

All data were analyzed using both STATA v14.2 (Stata Corp, College Station, TX, USA) and *R* v4.1.2 (R Project for Statistical Computing, Vienna, Austria). Confidence and intention Likert scale ratings were summarized as low (rating 0–3), moderate (4–6), or high (7–10). We used logistic regression to estimate associations between participant characteristics with preference for 1HP vs. 3HP. We used ordinal logistic regression to estimate associations between participants’ confidence and intention ratings (using ratings from 0 to 10) and their sociodemographic/clinical characteristics. As prior 3HP experience could impact preference for 1HP vs. 3HP, we analyzed the main outcomes excluding participants who had previously taken 3HP in sensitivity analyses.

### Ethical approval

This survey was approved by the School of Public Health Research Ethics Committee at the Makerere University College of Health Sciences (Kampala, Uganda), the Uganda National Council for Science and Technology (Kampala, Uganda), and the University of California San Francisco Institutional Review Board (San Francisco, CA, USA).

## RESULTS

**Figure 1.**
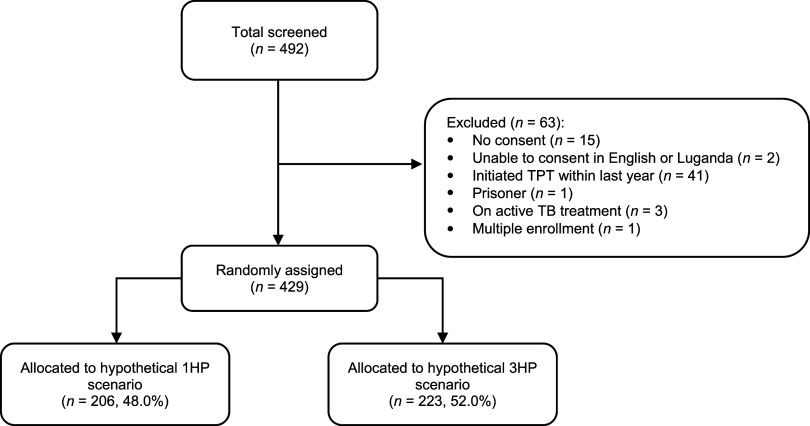
Summary of study participant eligibility screening and random assignment to the two hypothetical TB preventive treatment (TPT) scenarios, daily isoniazid-rifapentine for 1 month (1HP) or weekly isoniazid-rifapentine for 3 months (3HP).

In total, 492 PLHIV were screened, and 429 were included; 48% (206/429) were randomly assigned to the hypothetical 1HP scenario and 52% (223/429) to the hypothetical 3HP scenario (Figure 1). Participant characteristics are summarized in Table 1. Non-participants were similar in age and sex (data not shown).

**Table 1.** Participants’ sociodemographic and clinical characteristics, overall and by TPT scenario assignment.

		Random hypothetical TPT regimen	
	Overall	1HP	3HP
	(*n* = 429)	(*n* = 206)	(*n* = 223)
Characteristics	*n* (%)	*n* (%)	*n* (%)
Age, years, median [IQR]	43 [38–50]	43.5 [38–50]	43 [38–51]
Female	304 (70.9)	148 (71.8)	156 (70.0)
On hormonal contraception	60 (19.7)	35 (23.7)	25 (16.0)
Education level			
None	94 (21.9)	48 (23.3)	46 (20.6)
Primary	165 (38.5)	77 (37.4)	88 (39.5)
Secondary	126 (29.3)	60 (29.1)	66 (29.6)
Tertiary	44 (10.3)	21 (10.2)	23 (10.3)
Employed or self-employed	346 (80.6)	163 (79.1)	183 (82.1)
MPI^*^			
Not vulnerable	222 (52.0)	111 (53.9)	112 (50.2)
Vulnerable	122 (28.4)	60 (29.1)	62 (27.8)
Poor	70 (16.3)	27 (13.1)	43 (19.3)
Severely poor	14 (3.3)	8 (3.9)	6 (2.7)
ART regimen			
TDF/3TC/DTG	366 (85.3)	179 (86.9)	187 (83.9)
TDF/3TC/EFV	12 (2.8)	5 (2.4)	7 (3.1)
ABC/3TC/DTG	34 (7.9)	16 (7.8)	18 (8.1)
Other	17 (4.0)	6 (2.9)	11 (4.9)
Time on ART, years, median [IQR]	10.3 [7.2–14.2]	10.2 [7.4–14.3]	10.5 [6.9–14.0]
Suppressed viral load (<1,000 copies/ml)	423 (99.3)	204 (99.5)	219 (99.1)
Reported prior TB	77 (18.0)	40 (19.4)	37 (16.6)
Reported prior TPT	386 (90.0)	185 (89.8)	201 (90.1)
IPT	257 (66.6)	119 (64.3)	138 (68.7)
3HP	126 (32.6)	63 (34.1)	63 (31.3)
Both IPT and 3HP	3 (0.8)	3 (1.6)	0 (0.0)
Completed prior TPT	374 (97.0)	181 (98.0)	193 (96.0)
Side effects with prior TPT	96 (25.0)	43 (23.2)	53 (26.4)

* The MPI examines deprivations across 10 indicators in dimensions of health, education, and standards of living, with people deprived in one-third or more of all indicators considered to be multidimensionally poor. MPI values range from 0 to 1, with ‘1’ being the highest form of poverty; not vulnerable to poverty = MPI score <0.20; vulnerable to poverty = MPI score ≥0.20 but <0.33; poor = MPI score ≥0.33, but <0.50; severely poor = MPI score ≥0.50.

TPT = TB preventive treatment; 1HP = daily isoniazid-rifapentine for 1 month; 3HP = weekly isoniazid-rifapentine for 3 months; IQR = interquartile range; MPI = Multidimensional Poverty Index; ART = antiretroviral therapy; TDF = tenofovir; 3TC = lamivudine; DTG = dolutegravir; EFV = efavirenz; ABC = abacavir; IPT = daily isoniazid monotherapy (for 6 months).

Most study participants (71%, 304/429) were female, and the median age was 43 years (interquartile range [IQR] 38–50). Of the 429 participants, 335 (78%) had some education (39% primary school, 29% secondary school, 10% tertiary education) and most (81%, 346/429) were employed. The median time on ART was 10.3 years (IQR 7.2–14.2). Based on the MPI, 20% (84/429) of the participants were multidimensionally poor or severely poor. Most (90%, 386/429) had previously received TPT: either IPT (67%, 257/386), 3HP (32%, 126/386) or both (1%, 3/386). No other TPT regimens were reported. Adverse events with prior TPT were reported by 25% (96/386), more commonly among those on IPT.

**Figure 2.**
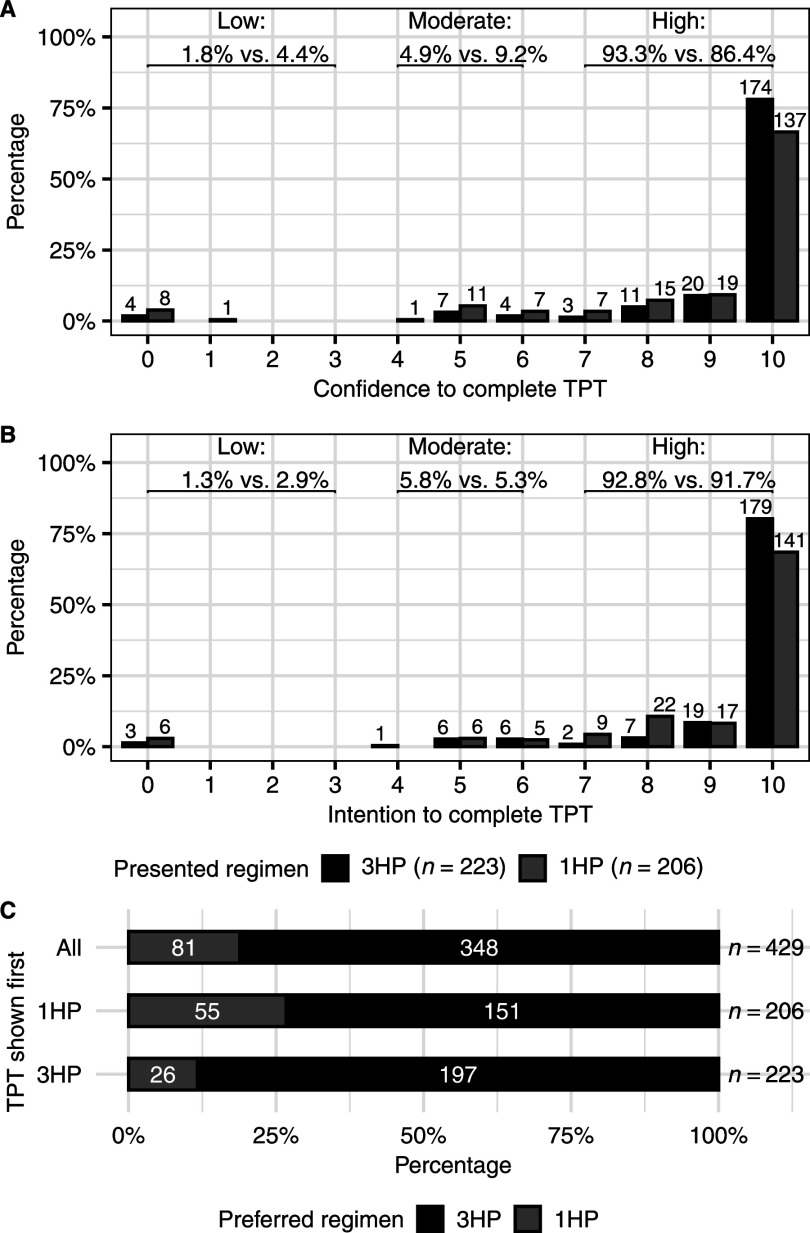
**A)** Participants’ level of perceived confidence and **B)** intention scores on an 11-point Likert scale ranging from 0 = strongly disagree to 10 = strongly agree, for completing the hypothetical TPT regimen to which they were randomized. The bars and corresponding numbers show the frequency of the Likert scale score for 3HP (black) or 1HP (grey). Percentages are shown in brackets at the top for the proportions of participants who expressed low (0–3), moderate (4–6) or high (7–10) confidence/intention for 3HP vs. 1HP. **C)** Participants’ preferences for 1HP vs. 3HP are shown for all participants and by regimen shown first at random assignment (1HP or 3HP). The bars indicate preference for 3HP (black) or 1HP (grey). TPT = TB preventive treatment; 1HP = daily isoniazid-rifapentine for 1 month; 3HP = weekly isoniazid-rifapentine for 3 months.

Both 1HP and 3HP were highly acceptable, and participants expressed high confidence to complete either 1HP (median Likert scale rating: 10, IQR 9–10) or 3HP (median rating: 10, IQR 10–10) (Figure 2). Confidence was rated high (7–10) by 86% (178/206) of participants for 1HP and 93% (208/223) for 3HP. Similarly, intention was rated high by 92% (189/206) of participants for 1HP and 93% (207/223) for 3HP. However, overall, most participants (81%, 348/429) preferred 3HP to 1HP, although preference differed according to the order of presenting the two regimens.

**Figure 3.**
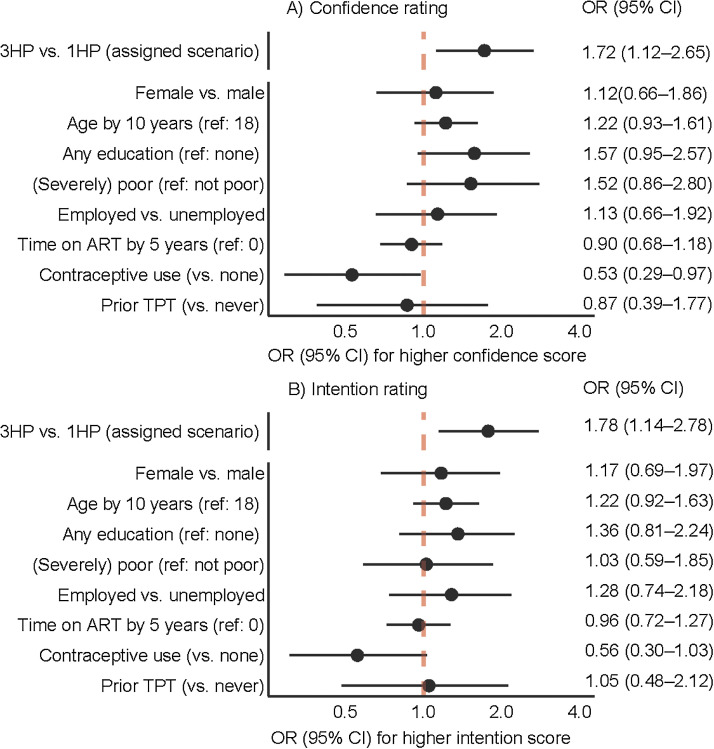
Variables associated with **A)** confidence and **B)** intention to complete TPT (n = 429 participants). The circle indicates the adjusted OR with a line to indicate the 95% CI. The vertical dotted line shows the null. The null denotes no association with confidence/intention. A circle to the right of the null whose line does not cross the null indicates higher confidence/intention. A circle to the left of the null whose line does not cross the null indicates lower confidence/intention. A circle whose line crosses the null indicates no association with confidence/intention. The ORs compare subgroups of assigned hypothetical TPT scenario, 3HP vs. 1HP, as well as baseline variables. Multidimensional poverty was included as a bivariate: we compared the group which was poor or severely poor to the reference group which was defined as vulnerable or not vulnerable (i.e., not poor). TPT = TB preventive treatment; OR = odds ratio; CI = confidence interval; 3HP = weekly isoniazid-rifapentine for 3 months; 1HP = daily isoniazid-rifapentine for 1 month; ART = antiretroviral therapy.

In an ordinal logistic regression using the 0–10-point Likert scale ratings, confidence was rated higher by those randomized to the hypothetical 3HP regimen compared to the hypothetical 1HP regimen (adjusted odds ratio [aOR] 1.72, 95% confidence interval [CI] 1.12–2.65). Participants using hormonal contraception rated their confidence lower than those who were not using hormonal contraception (aOR 0.53, 95% CI 0.29–0.97). Confidence ratings did not differ by age, sex, education status, MPI category, employment status, time on ART or prior TPT (Figure 3A). In a sensitivity analysis excluding participants with prior 3HP experience, results were similar (Supplementary Figure S1A).

In the ordinal logistic regression for the 0–10-point Likert scale rating of intention, randomization to the hypothetical 3HP regimen was associated with higher intention to complete treatment (aOR 1.78, 95% CI 1.14–2.78). Intention ratings did not differ by age, sex, education status, MPI category, contraceptive use, employment status, time on ART or prior TPT (Figure 3B). Results were similar in a sensitivity analysis excluding participants with prior 3HP experience (Supplementary Figure S1B).

After adjusting for other variables, the participant groups most likely to prefer 1HP were those assigned to the hypothetical 1HP scenario (27% preferred 1HP over 3HP: aOR 3.01, 95% CI 1.78–5.08) and those with no history of prior TB (21% preferred 1HP over 3HP: aOR 3.27, 95% CI 1.32–8.08). In no group did more than 27% of participants prefer 1HP over 3HP (Table 2). Participants’ preferences for 1HP vs. 3HP did not differ by prior TPT experience. Our findings on participants’ preferences for 1HP vs. 3HP did not change significantly in the sensitivity analysis that excluded participants with prior 3HP experience (Supplementary Table S1).

**Table 2.** Variation in preference for 1HP vs. 3HP by participants’ characteristics.

		Participants preferring 1HP	Preference for 1HP*		Preference for 1HP*	
Characteristic	*N*	*n* (%)	OR (95% CI)	*P*-value	aOR (95% CI)	*P*-value
Overall preference	429	81 (18.9)				
Age group (median split), years						
18–43 (reference)	216	49 (22.7)				
≥44	213	32 (15.0)	0.60 (0.37–0.99)	0.04	0.63 (0.37–1.05)	0.08
Sex						
Male (reference)	125	20 (16.0)				
Female	304	61 (20.1)	1.32 (0.76–2.29)	0.33	1.16 (0.64–2.10)	0.63
Employment status						
Unemployed (reference)	83	13 (15.7)				
Employed	346	68 (19.7)	1.32 (0.69–2.52)	0.41	1.54 (0.77–3.06)	0.22
Prior TB						
Yes (reference)	77	6 (7.8)				
No	352	75 (21.3)	3.20 (1.34–7.66)	0.009	3.27 (1.32–8.08)	0.01
Prior TPT^†^						
Never (reference)	43	8 (18.6)				
IPT	257	54 (21.0)	1.16 (0.51–2.66)	0.72	0.87 (0.36–2.10)	0.76
3HP	129	19 (14.7)	0.76 (0.30–1.88)	0.55	0.55 (0.21–1.43)	0.22
Assigned TPT scenario						
3HP (reference)	223	26 (11.7)				
1HP	206	55 (26.7)	2.76 (1.65–4.61)	<0.001	3.01 (1.78–5.08)	<0.001

* Preference for 3HP is the reference group.

^†^ Participants who had taken both IPT and 3HP are included in the 3HP group.

OR = odds ratio; CI = confidence interval; aOR = adjusted OR; TPT = TB preventive treatment; IPT = daily isoniazid monotherapy (for 6 months); 3HP = weekly isoniazid-rifapentine for 3 months; 1HP = daily isoniazid-rifapentine for 1 month.

## DISCUSSION

In this cross-sectional survey of 429 PLHIV randomly assigned to hypothetical TPT scenarios of receiving 1HP or 3HP at a large urban HIV/AIDS clinic in Kampala, Uganda, we found that 3HP was more often preferred, but both regimens were highly acceptable. When given a choice, over 80% preferred 3HP (88% of those randomized to the hypothetical 3HP regimen, 73% of those randomized to hypothetical 1HP). These findings suggest that health systems should elicit PLHIV preferences before scaling-up both 3HP and 1HP, and that in Uganda, greater focus should be on 3HP.

Acceptability was high for both regimens, indicating that either regimen, if offered as the only option, would likely yield high TPT coverage. This high acceptability is consistent with findings of high treatment acceptance and completion for both regimens demonstrated by several randomized clinical trials.^6,8,22,23^

Although shorter treatments are often assumed to be preferred, we found the opposite in this study – perhaps reflecting preferences for fewer medication days or lower pill burden. We informed participants that 1HP may require adjusting their daily ART dosage by adding an extra dolutegravir pill to cater for drug–drug interactions with rifapentine.^24^ Moreover, weekly dosing may be preferred over daily dosing. A qualitative study in Peru found that participants perceived less frequent dosing as less toxic.^25^ Although we presented potential side effects as equivalent for both regimens, this perception could have influenced our participants’ choice.

In addition, preference for 3HP might also reflect greater familiarity with the regimen, although we did not find an association with prior TPT experience and preference. About one third of participants had previously taken 3HP, most of them likely in fixed-dose combinations. However, participants may have been familiarized with 3HP indirectly through the experiences of peers at the clinic, through its rollout in a randomized trial,^19^ and programmatically through the HIV program,^5^ or via the clinic’s routine health education talks.

No prior studies have directly compared the preferences of adult PLHIV for 1HP vs. 3HP. In pediatric populations, reported drivers of TPT preference were pill burden (treatment duration and frequency), medication fatigue due to prolonged daily dosing, and capacity to remember to take their medicines.^26^ 3HP was deemed more preferable to IPT due to its weekly dosing schedule and minimal disruption to daily lives.^25^ Qualitative evidence from the Mulago ISS clinic collected prior to the roll-out of 3HP, demonstrated that weekly dosing was a key facilitator for patient acceptance and completion of 3HP.^27^

High acceptability of a short-course TPT regimen is key to achieving public health goals. Although we found that 3HP was associated with higher confidence and intention, both regimens were highly acceptable (regardless of patient characteristics, including education), and it is unclear whether small absolute differences of confidence or intention would translate to a relevant increase in TPT coverage and treatment completion compared to 1HP. These differences may also be affected by scale biases, where some groups may be more or less likely to select the end of the scale.

Our study has some limitations. First, although being offered TPT was a realistic scenario for this population, the scenarios were nevertheless hypothetical. Observation of real choices (i.e., revealed preferences) under actual treatment implementation would provide more direct evidence of patient preference for 1HP vs. 3HP. In addition, our measurement of preferences using a quantitative scale may not have been contextually suitable to the diverse population of HIV-positive Ugandans, with varying literacy gaps and sociocultural influences. However, this survey was administered by experienced research staff, both TPT scenarios were well explained, and piloting suggested that all elements of the study were well understood, including the quantitative scale. Finally, this survey was done in an urban, highly ART-experienced population. Future studies may consider replicating this assessment in populations with less ART literacy and in rural settings with less TPT experience.

In summary, this study highlighted that both 1HP and 3HP were highly acceptable TPT regimens among 429 PLHIV in Kampala, Uganda, with 3HP being the preferable option to many, despite the common assumption that its longer duration would make it less preferable. Our findings suggest that health systems should not simply replace 3HP with 1HP based on the untested perception that a regimen of shorter duration will necessarily be more acceptable or result in higher levels of completion. These findings strongly support the roll-out of short-course TPT (both 3HP and 1HP) in high TB-HIV burden settings. Where feasible, patients should be given choices between regimens; in Uganda, if providing such choice is not feasible, priority may be given to 3HP.

## Supplementary Material


