# Supplementary material for: Preference for daily (1HP) vs. weekly (3HP) isoniazid-rifapentine among people living with HIV in Uganda
Source: IJTLD Open. 2024 Feb 1;1(2):83–9. doi: 10.5588/ijtldopen.23.0283 (PMC11221590; doi:10.5588/ijtldopen.23.0283)
Supplement: Supplementary file 1 [file iutld_ijtld_open_23.0283_supplementarydata1.pdf]

Supplementary Data 1: Participant education

NOTE: This introduction is intended for all participants. Participant facing slides have been removed from this version but are shown in the inset picture.

Part 1:Introduction to Tuberculosis (TB), Latent TB Infection (LTBI) and TB Preventive Treatment (TPT)

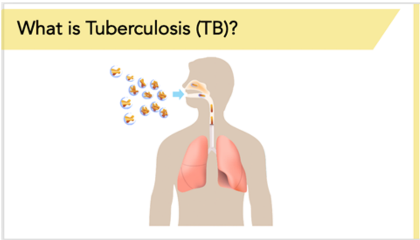

- TB is a disease caused by a germ that attacks the lungs. It can attack other parts of the body.
- TB germs are inhaled by an uninfected person. The healthy immune system can stop the germs from spreading leading to latent TB infection (**LTBI;** “sleeping TB”).
- If the immune system is weakened, the TB germs will replicate and cause disease (“active TB”).

1

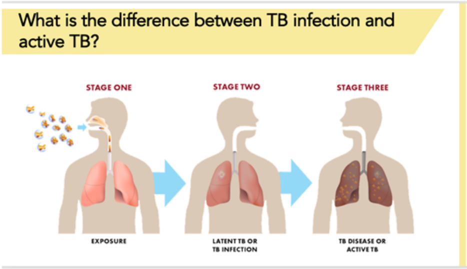

- People who become infected with TB germs, but do not feel sick have latent (“sleeping”) TB.
- This form is not contagious.
- If left untreated, sleeping TB can develop into active TB disease, the type of TB that makes you sick and can be spread to others.

2

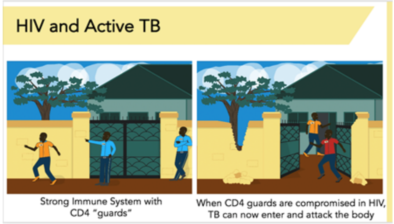

Make sure the participant understands that the walls and the gate represent the immune system. The guards represent their CD4 count. HIV makes it so that the guards of the immune system (CD4 count) are compromised, making it easier for TB infection to become active TB and enter the system, making a person get sick.

- While anyone can develop TB, people living with HIV are at HIGH RISK of getting active TB.
- The immune system prevents a person from getting sick. HIV can weaken the immune system.
- Taking antiretroviral therapy (ARVs) helps to keep the body strong and also reduces the chances of getting active TB.
- Being infected with TB puts people living with HIV at risk of becoming very sick and even dying.
- Sleeping TB can become active because of HIV.

3

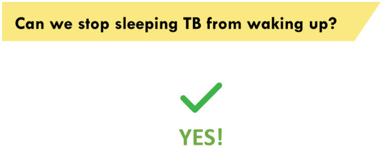

- We can prevent “sleeping TB” from waking up using preventive medicine.
- These medicines kill the germs in the body and stop them from waking up to cause active TB in the future.
- These medicines can also prevent infection with sleeping TB in case you have not yet been infected.

4

Questions?

Do you have any questions about active TB, sleeping TB or the medicines used to stop sleeping TB from waking up?

Before you continue:

1. Make sure you answer any participant questions or concerns.
2. The participant is allowed to go back to the explanations of active TB, sleeping TB and TPT, the medicines used to prevent sleeping TB from waking up, during the survey.

READ ALOUD:

- I'm now going to talk to you about some of these medicines used to prevent TB.
- We are not going to give you these medicines at this time. We are only interested in your thoughts about these medicines.

5

What is 1HP made of?

- 1HP is made of two medicines that kill TB germs
- These two medicines are called:
  1. Rifapentine
  2. Isoniazid

- 1HP is made of two medicines combined that kill TB germs.
- These two medicines are called:
  1. Rifapentine
  2. Isoniazid
- One dose of 1HP is made up of two parts of TB medicines that kill TB germs.
- One part of the 1HP dose is taken using a combined tablet containing both rifapentine and isoniazid.
- The other part of the 1HP dose is taken using separate tablets containing only rifapentine.

6

Part 2:

Version B: 1HP Information

NOTE: This section is intended to provide a brief description of the 1HP regimen to all participants randomized to the hypothetical 1HP regimen prior to eliciting their level of perceived confidence and intention to complete this regimen.

How many tablets would you have to take if you were given 1HP?

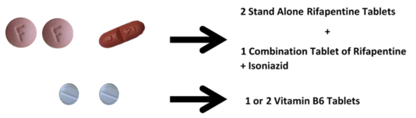

- A dose of 1HP involves swallowing **four or five** tablets:
- **3 tablets** contain medicines that kill TB germs. These are:
  - 2 tablets of rifapentine alone
  - 1 tablet of rifapentine combined with isoniazid
- One dose of 1HP also contains **one or two** tablets of vitamin B6; depending on using 25mg or 50mg tablets.
- Vitamin B6 is used to prevent side effects of taking the TB medicines.

7

How often would you have to take these four or five tablets?

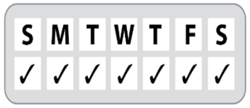

- You would take these four or five tablets daily.

8

How long is 1HP treatment?

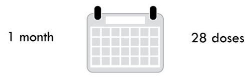

- You would have to take four or five tablets, every day for one month to complete 1HP treatment.
- Since each month is made up of four weeks, that means that you would take four or five tablets every day for 4 weeks.
- Therefore, to complete treatment, you would have to take 28 doses.

9

Is it safe to take 1HP with your ARVs?

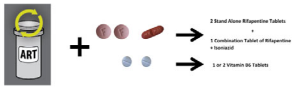

- Yes, most HIV tablets are safe to take with 1HP, but some are not.
- For people who are taking ARV tablets that interact with 1HP, the most likely change to the ARV regimen will be to add one tablet to the ARV treatment to increase the dose.
- It is very important to remember that we are not going to ask you to take 1HP today; we are only interested in your thoughts about these medicines.
- If you were given these tablets, you would have to also continue taking your daily ARV tablets.

10

Can 1HP affect any other medicines that you may be taking besides ARVs?

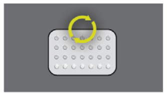

Yes!!

- Yes, 1HP decreases the effectiveness of certain drugs such as anti-malarial drugs and antibiotics used to treat illnesses like pneumonia.
- For women, 1HP decreases the effectiveness of hormonal contraceptives such as the three-month injection (depo/sayana press), monthly pills (microgynon) or arm implants for three or five years (implanon/jadelle).
- Women must use additional non-hormonal contraception such as condoms to avoid getting pregnant while taking 1HP.

11

Are there side effects with these medicines?

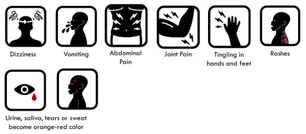

- Most people do not get side effects because these medicines are generally very safe. But like with all medicines, there is a small chance that people taking 1HP may have side effects.
- The commonest side effects may include:
  - Nausea, vomiting, or abdominal pain,
  - Flu-like symptoms
  - Dizziness or tiredness,
  - New joint pains, or
  - Skin rashes
  - This medicine may sometimes cause tingling and numbness in the hands and feet. Vitamin B6 is given to prevent this.
- It is *common* when taking these tablets that urine, saliva, tears, or sweat becomes an orange-red color. This is normal and will go back to normal when the month of treatment is completed.

12

Questions?

Do you have any questions about 1HP?

Before you continue:

1. Make sure you answer any participant questions or concerns.
2. The participant is allowed to go back to the explanations of the features of the 1HP regimen during the survey.

Note for study staff:
Please proceed to rating the participant's level of perceived confidence and intention to complete 1HP in part three.

13

What is 3HP made of?

- 3HP is made of two medicines combined together that kill TB germs
- These two medicines are called:
  1. Rifapentine
  2. Isoniazid

- 3HP is made of two medicines combined together that kill TB germs
- These two medicines are called:
  1. Rifapentine
  2. Isoniazid
- These two medicines are combined together in the same tablets.

14

Part 2

Version B: 3HP Information

NOTE: This section is intended to provide a brief description of the 3HP regimen to all participants randomized to the hypothetical 3HP regimen prior to eliciting their level of perceived confidence and intention to complete this regimen.

How many tablets would you have to take if you were given 3HP?

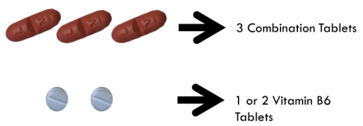

A dose of 3HP includes swallowing **four or five tablets:**

- **3 tablets** that contain the medicines that kill TB germs, and
- **1 or 2 tablets** of vitamin B6 (depending on using 25mg or 50mg tablets)
- Vitamin B6 is used to prevent side effects of taking the TB medicines.

15

How often would you have to take these four or five tablets?

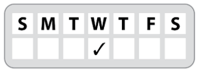

- You would take these four or five tablets once a week.
- You would choose one day a week and always take these tablets on that day at the same time every week until you complete the full regimen.
- For example, you may choose Wednesday to take your tablets. You would take these tablets every Wednesday at the same time until you complete.

16

How long is 3HP treatment?

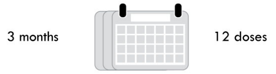

- You would have to take four or five tablets, once a week for three months to complete the full 3HP regimen.
- Since each month is made up of four weeks, that means that you would take four or five tablets once a week for 12 weeks.
- To complete treatment, you would have to take 12 doses.

17

Is it safe to take 3HP with your ARVs?

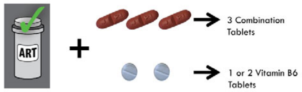

- Yes, most HIV tablets are safe to take with 3HP, but some are not.
- It is very important to remember that we are not going to ask you to take 3HP today; we are only interested in your thoughts about these medicines.
- If you were given these tablets, you would have to also continue taking your daily ARV tablets.

18

Can 3HP affect any other medicines that you may be taking besides ARVs?

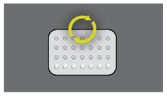

Yes!!

- Yes, 3HP decreases the effectiveness of certain drugs such as anti-malarial drugs and antibiotics used to treat illnesses like pneumonia.
- For women, 3HP decreases the effectiveness of hormonal contraceptives such as the three-month injection (depo/sayana press), monthly pills (microgynon) or arm implants for three or five years (implanon/jadelle).
- Women must use additional non-hormonal contraception such as condoms to avoid getting pregnant while taking 3HP.

19

Are there side effects with these medicines?

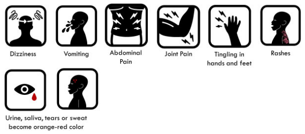

- **Most people** do not get side effects because these medicines are generally very safe. But like with all medicines, there is a small chance that people taking 3HP may have side effects.
- The commonest side effects may include:
  - Nausea, vomiting, or abdominal pain,
  - Flu-like symptoms
  - Dizzy or tiredness,
  - New joint pains, or
  - Skin rashes
- This medicine may sometimes cause tingling and numbness in the hands and feet. Vitamin B6 is given to prevent this.
- It is *common* when taking these tablets that urine, saliva, tears, or sweat becomes an orange-red color. This is normal and will go back to normal when the 12 weeks of treatment are completed.

20

Questions?

Do you have any questions about 3HP?

Before you continue:

- Make sure you answer any participant questions or concerns.
- The participant is allowed to go back to the explanations of the features of the 3HP regimen during the survey.

Note for study staff:
Please proceed to rating the participant's level of perceived confidence and intention to complete 3HP in part three.

21

I feel confident that I would be capable of completing all 28 daily doses of this treatment.

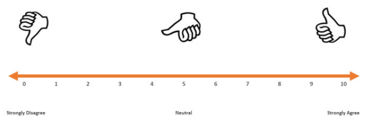

INSTRUCTIONS FOR STUDY STAFF:

- Ask the participant to rate his/her level of confidence to complete all the 28 daily doses of 1HP using the Likert scale from 0 to 10.
- 0 means the participant is not confident at all and therefore strongly disagrees.
- 5 means the participant is not sure and is therefore neutral.
- 10 means the participant is very confident and therefore strongly agrees.

22

Part 3

Version A: Perceived Confidence and Intention to Complete 1HP

NOTE: This section is intended to help participants rate their levels of perceived confidence and intention to complete the 28 daily doses of 1HP treatment using a 10-point Likert scale.

I would intend to complete all 28 daily doses of this treatment.

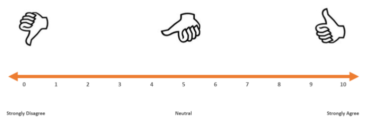

INSTRUCTIONS FOR STUDY STAFF:

- Ask the participant to rate his/her level of intention to complete all the 28 daily doses of 1HP using the Likert scale from 0 to 10.
- 0 means the participant would not intend to at all and therefore strongly disagrees.
- 5 means the participant is not sure and is therefore neutral.
- 10 means the participant would strongly intend to and therefore strongly agrees.

Note for study staff:
Please proceed to providing the participant with a brief comparison between 1HP and 3HP in part four.

23

Part 3

Version B: Perceived Confidence and Intention to Complete 3HP

NOTE: This section is intended to help participants rate their levels of perceived confidence and intention to complete the 12 weekly doses of 3HP treatment using a 10-point Likert scale.

I feel confident that I would be capable of completing all 12 weekly doses of this treatment.

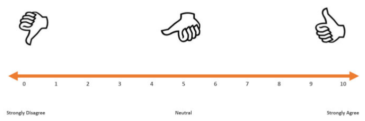

INSTRUCTIONS FOR STUDY STAFF:

- Ask the participant to rate his/her level of confidence to complete all the 12 weekly doses of 3HP using the Likert scale from 0 to 10.
- 0 means the participant is not confident at all and therefore strongly disagrees
- 5 means the participant is not sure and is therefore neutral
- 10 means the participant is very confident and therefore strongly agrees

24

I would intend to complete all 12 weekly doses of this treatment.

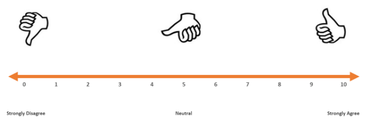

INSTRUCTIONS FOR STUDY STAFF:

- Ask the participant to rate his/her level of intention to complete all the 12 weekly doses of 3HP using the Likert scale from 0 to 10.
- 0 means the participant would not intend at all and therefore strongly disagrees.
- 5 means the participant is not sure and is therefore neutral.
- 10 means the participant would strongly intend to and therefore strongly agrees.

Note for study staff:
Please proceed to providing the participant with a brief comparison between 3HP and 1HP in part four.

25

Part 4: 1HP vs. 3HP

NOTE: We are now going to tell you about another regimen for TB prevention called 3HP and how it compares to 1HP.

The 1HP regimen involves swallowing 4 or 5 tablets daily for 1 month with a total of 28 doses.

1HP vs. 3HP?

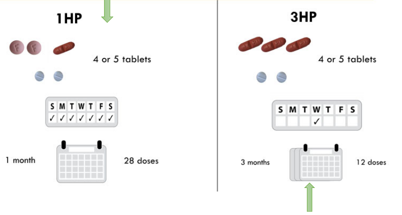

The 3HP regimen involves swallowing 4 or 5 tablets, once-a-week, for 3 months with a total of 12 doses.

- The same medicines are used in the 3HP regimen as those of 1HP.
- Both 1HP and 3HP involve swallowing 4 or 5 tablets per dose.
- 3HP tablets are swallowed once-a-week unlike the daily dosing of 1HP.
- The total number of doses is lower for 3HP (12) compared to 1HP (28).
- The duration of treatment is longer for 3HP (3 months) compared to 1HP (1 month)

26

1HP vs. 3HP?

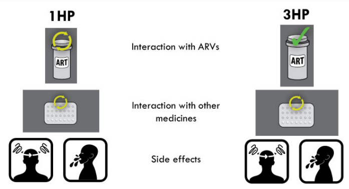

- Both 1HP and 3HP are safe to use with most ARVs.
- However, with 1HP, your daily dose of ARV tablets may be increased by one tablet.
- Your daily dose of ARV tablets does not change with 3HP.
- Both 1HP and 3HP reduce the effectiveness of other medicines such as antimalarial drugs.
- Both 1HP and 3HP have similar side effects.

Note for study staff:

- Make sure you answer any participant questions or concerns.
- Then proceed to asking the participant for his/her preference between 1HP and 3HP on the next slide.

27

Which treatment option would you prefer between 1HP and 3HP?

If you were given the choice between these two treatment options to prevent TB, which would you prefer?

INSTRUCTIONS FOR STUDY STAFF:

- Ask the participant to state what his/her preferred treatment option for preventing TB would be between 1HP and 3HP using the following statement:
- **If you were given the choice between these two treatment options to prevent TB, which would you prefer?**

28

Supplementary Table S1 Variation in participants' preferences for 1HP compared to 3HP by participants' characteristics – sensitivity analysis results, excluding 129 participants with prior 3HP experience

**Characteristic**	**N**	**Participants preferring 1HP[1] n (%)**	**Unadjusted OR, Preference for 1HP (95% CI)**	**p-value**	**Adjusted OR, Preference for 1HP (95% CI)**	**p-value**
**Overall preference**	300	62 (20.7)				
**Age-group (median split)**						
18-43 years (reference)	152	39 (25.7)				
≥44 years	148	23 (15.5)	0.53 (0.30-0.95)	0.03	0.58 (0.32-1.05)	0.07
**Sex**						
Male (reference)	87	15 (17.2)				
Female	213	47 (22.1)	1.36 (0.71-2.59)	0.35	1.08 (0.54-2.18)	0.82
**Employment status**						
Unemployed (reference)	64	12 (18.7)				
Employed	236	50 (21.2)	1.17 (0.58-2.35)	0.67	1.37 (0.65-2.88)	0.41
**Prior Tuberculosis**						
Yes (reference)	51	4 (7.8)				
No	249	58 (23.3)	3.57 (1.23-10.32)	0.02	3.84 (1.27-11.60)	0.02
**Prior TPT**						
Never (reference)	43	8 (18.6)				
IPT	257	54 (21.0)	1.16 (0.51-2.66)	0.72	0.85 (0.35-2.06)	0.71
**Assigned TPT scenario**						
3HP (reference)	160	22 (13.7)				
1HP	140	40 (28.6)	2.51 (1.40-4.48)	0.002	2.68 (1.47-4.88)	0.001

1HP = daily isoniazid-rifapentine for one month, *3HP* = weekly isoniazid-rifapentine for three months, *OR* = odds ratio, *CI* = confidence interval, *TPT* = tuberculosis preventive treatment

1. *Preference for 3HP is the reference group*

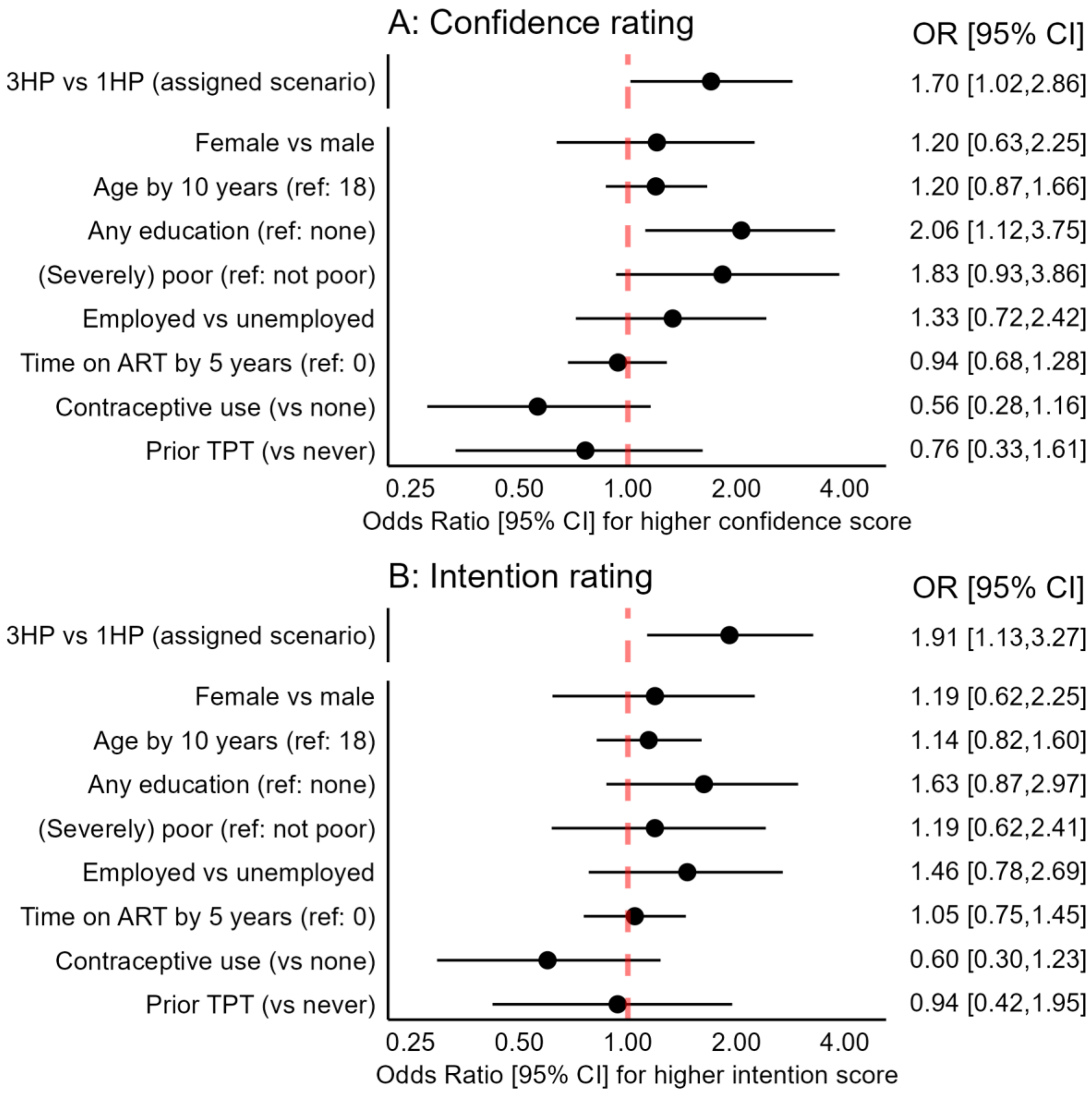

Supplementary Figure S1 *Sensitivity analysis of associations of confidence and intention ratings with assigned TPT scenario and baseline variables, estimated in an ordinal logistic regression for* **A)** *higher confidence ratings on a Likert scale of 0 to 10, and* **B)** *higher intention ratings on a Likert scale of 0 to 10. This analysis included 300 participants, excluding 129 participants with prior 3HP experience.*

The circle indicates the adjusted odds ratio with a line to indicate the 95% confidence interval. The vertical dotted line shows the null. The odds ratios compare subgroups of assigned hypothetical TB preventive treatment (TPT) scenario (3HP vs. 1HP), as well as baseline variables. Multidimensional poverty was included as a bivariate: we compared the group which was poor or severely poor to the reference group which was defined as vulnerable or not vulnerable (i.e., not poor).
